# Harnessing Small RNAs as Synthetic Post-transcriptional Regulators in Bacteria

**DOI:** 10.1021/acssynbio.5c00118

**Published:** 2025-07-08

**Authors:** Jens Georg, Bork A. Berghoff, Daniel Schindler

**Affiliations:** † Institut für Biologie III, 9174 Albert-Ludwigs-Universität Freiburg, Schänzlestraße 1, 79104 Freiburg, Germany; ‡ Institute of Molecular Biology and Biotechnology of Prokaryotes, 9189 University of Ulm, Albert-Einstein-Allee 11, 89081 Ulm, Germany; § 28310 Max Planck Institute for Terrestrial Microbiology, Karl-von-Frisch-Straße 10, 35043 Marburg, Germany; ∥ Center for Molecular Biology of Heidelberg University (ZMBH), INF 329, 69120 Heidelberg, Germany; ⊥ Center for Synthetic Microbiology, Philipps-University Marburg, Karl-von-Frisch-Straße 14, 35032 Marburg, Germany

**Keywords:** synthetic biology, small regulatory RNA, seed region, scaffold, post-transcriptional regulation, biotechnology

## Abstract

Bacteria can respond to environmental changes by expressing small RNAs (sRNAs), which regulate mRNAs by complementary base-pairing. This regulatory mechanism allows bacteria to rapidly adapt their proteome. In recent years, sRNAs have gained attention as blueprints for synthetic regulators allowing control over user-defined targets. Multiple aspects need to be considered for efficient application of these versatile, on-demand, and easy-to-use tools. Advances in computational prediction and bioengineering concepts are the dawn of systematic synthetic sRNA biology. We provide an overview of sRNAs and alternative post-transcriptional regulators, highlight the requirements for successful regulation, and provide guidelines for design, construction, and sRNA application.

## Graphical abstract

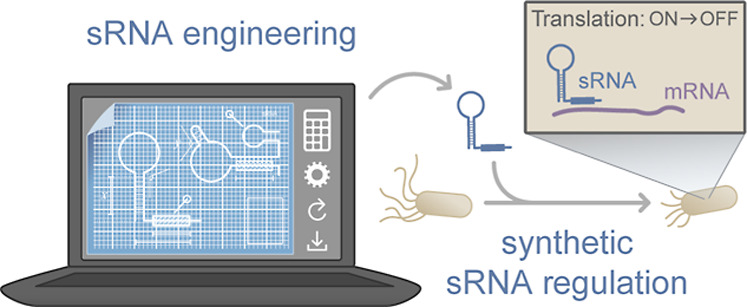

## An Introduction to RNA-Based Regulation in Bacteria

Synthetic biologists see living systems from an engineer’s perspective, and standardization and characterization are fundamental elements with one of the ultimate goals to be able to turn cells into highly controlled systems. Nature has invented numerous types of RNA-based regulation, many of which are promising blueprints for the design of synthetic regulators. Their adaptation as versatile, modular, on-demand tools in prokaryotes is currently the topic of intense investigations. The versatility of RNA-based regulators is reflected by the many modes of action: depending on the particular system and the auxiliary factors, target genes may be regulated at the level of transcription, translation, or mRNA stability (Figure 1). Here, we give a brief overview of RNA-based regulators before focusing on small regulatory RNAs as post-transcriptional regulators.

**Figure 1:**
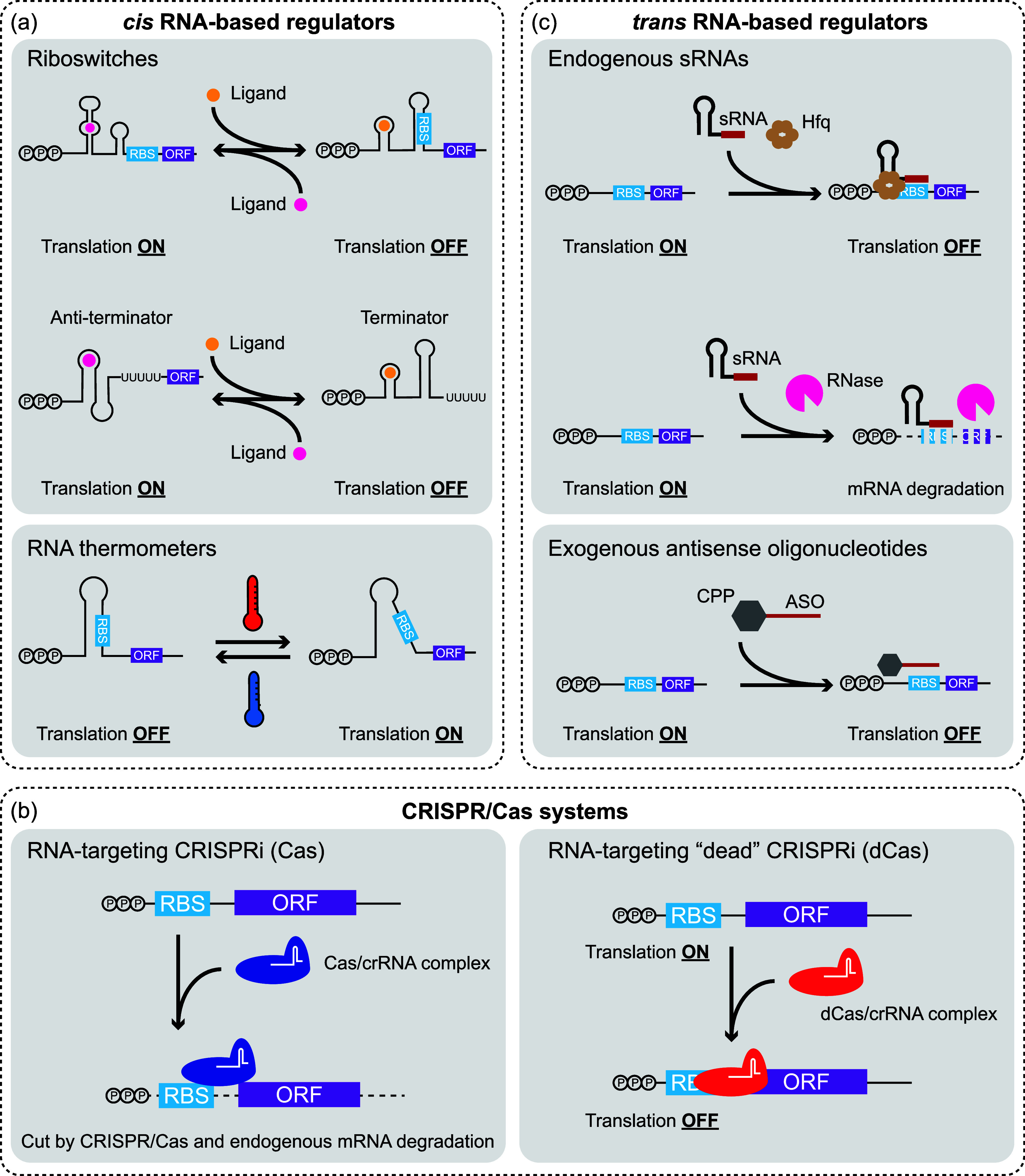
RNA-based systems for gene regulation in bacteria. (a) *cis* RNA-based regulators (riboswitches and RNA thermometers) are located in the 5′ UTR of an mRNA. Upon binding of a ligand, riboswitches undergo a conformational change, resulting in translation activation/repression or transcription termination/antitermination. RNA thermometers change their conformation in response to a temperature change. (b) Possible applications of RNA-targeting CRISPR/Cas systems are destabilization of mRNAs using an RNA-targeting Cas endonuclease (e.g., Cas13, Cas7–11) and a CRISPR RNA (crRNA) or translation inhibition by an endonuclease-deficient, RNA-targeting Cas. RBS: ribosome binding site, ORF: open reading frame. (c) Small RNAs (sRNAs) are endogenously produced and—with the help of RNA chaperones or RNases—either block translation or destabilize mRNAs. Delivery of antisense oligonucleotides (ASOs) to prokaryotic cells is aided by cell-penetrating peptides (CPPs). ASOs block translation of target mRNAs.

RNA-based regulators can be divided into intrinsic (*cis*-acting) and extrinsic (*trans*-acting) mechanisms. Intrinsic regulators are mostly inherent to the 5′ untranslated region (UTR) of the mRNA which they regulate. Their mode of action is a two-step process, consisting of “sensing” and “regulation”. In response to a cellular or environmental signal, the regulatory RNA sequence undergoes a conformational change (sensing) that affects downstream gene expression typically at the level of transcription termination or translation (regulation). In the case of riboswitches, the RNA aptamer region binds to a ligand, followed by structural rearrangements of the expression platform, resulting in regulation of the downstream gene(s) (Figure 1a). Riboswitches selectively bind many different ligands, such as small molecules and elemental ions,^1,2^ resulting in either positive or negative regulation. These features turn riboswitches into versatile tools for synthetic biology applications.^3^ However, the development of synthetic riboswitches typically involves the laborious screening of randomized libraries in a somewhat “plug-and-pray”-like approach.^4^ Efforts to generate functional riboswitches in a “plug-and-play” fashion based on rational design are becoming increasingly successful,^5^ but will certainly benefit from advances in machine learning (ML) and artificial intelligence-based (AI) approaches in the future. The basic principles of RNA thermometers are similar to riboswitches. However, instead of ligands, RNA thermometers change their conformation in response to a temperature change. This mode of action is inherent to the mRNAs of many heat shock and virulence genes in bacteria.^6^ Even though most RNA thermometers primarily regulate translation initiation (Figure 1a), regulation of transcription termination is also feasible and may be exploited for synthetic biology approaches.^7^ Finally, riboswitches and RNA thermometers can be combined to generate novel *cis*-regulators that allow the modulation of gene expression by both ligands and temperature, respectively.^8^

The prokaryotic immune system CRISPR/Cas has been extensively studied and applied as a genome editing tool in both eukaryotes and prokaryotes.^9^ The endonuclease-deficient Cas proteins (dCas) can be used to inhibit or activate transcription resulting in “RNA-programmable transcription factors”.^10^ Interestingly, natural Cas systems silencing gene expression in targeting regions also exist.^11,12^ There are also CRISPR/Cas systems targeting RNA (e.g., Cas13,^13^ Cas7–11 ^14^), which may be applied as post-transcriptional regulators^15^ (Figure 1b). But it is documented that the RNA-targeting CRISPR systems induce dormancy or cell death in bacteria, naturally preventing the spread of phages within a population. Cas-complexes rely on the combination of proteins and RNAs and thus require the delivery of large DNA cargos to cells potentially resulting in cellular burden. Furthermore, dCas systems have shown toxic effects due to their tight binding to DNA.^16–18^ It has even been demonstrated that CRISPR/dCas can prevent initiation of DNA replication if the origin of replication is targeted.^19^ This can lead to cell death if the complex does not dissociate.

This article focuses on extrinsic RNA-based regulators of the small regulatory RNA (sRNA) group, which often rely on (endogenous) auxiliary factors, such as RNA chaperones and RNases, to fulfill their regulatory actions. Naturally occurring sRNAs have sizes of 50–100 nucleotides, but longer sRNAs, which can contain small open reading frames, have also been described.^20^ They are typically expressed under stress conditions and use a relatively short stretch of nucleotides, the so-called seed region, for imperfect base-pairing with their target mRNAs.^21–23^ Binding of targets may be facilitated by RNA chaperones, such as Hfq (see box [Boxed-text box1]) or ProQ, and ultimately inhibit translation due to blockage of the ribosome binding site (RBS) (Figure 1c). Alternatively, mRNAs are destabilized due to the recruitment of RNases or transcription is modulated by the promotion of Rho-dependent termination^24,25^ or antitermination.^26^ While sRNAs are produced endogenously by the cell, this system serves as a blueprint for the generation of exogenously applied programmable regulators/therapeutics. Most known natural sRNA interactions have a repressive effect, but there are also many examples of activation by sRNAs (translation activation, antitermination, sponging of negative regulators, repression of negative regulators, RNase protection).^27^ In general, activating mechanisms require a pre-existing negative factor or mechanism (e.g., termination site, inhibitory secondary structure, processing site etc.) that can be counteracted by an sRNA. If these are unknown or nonexistent for a gene of interest it is hard to utilize synthetic sRNAs for activation. However, genetically engineered Rho-independent terminator hairpins with sRNA-tunable folding have been successfully applied in conjunction with designed Small Transcription Activating RNAs (STARs).^28,29^ Antisense oligonucleotides (ASOs)^30,31^ may be administered from the outside to penetrate the bacterial cell and repress mRNAs by base-pairing, resulting in translation inhibition. ASOs are short stretches of nucleic acid analogs, such as peptide nucleic acids (PNAs), with an increased stability and RNA-binding affinity compared to regular nucleic acids.^32^ For their delivery into bacterial cells, ASOs are commonly conjugated to cell-penetrating peptides (CPPs) (Figure 1c). While ASO–CPP conjugates hold promise as programmable antibiotics by targeting essential genes of pathogenic bacteria,^30,33^ they can also be exploited in synthetic biology to reprogram gene expression.^34^ One disadvantage is that they must be administered externally, as they cannot be produced heterologously in vivo. For this reason, this article only briefly covers ASOs. Nevertheless, they can be screened in a high-throughput cell-free system and utilized as an sRNA antagonist.^35^

1 **The RNA chaperone Hfq.**

Hfq is the prototype of an RNA chaperone. First identified as a host factor for phage infection, it was later shown that Hfq enhances the stability of some sRNAs and facilitates sRNA-based regulation (reviewed in^36^). Hfq forms a homohexamer with three RNA-binding faces. The proximal site binds U-rich RNA stretches, the rim binds UA-rich sequences, and the distal Hfq site binds ARN-repeats (A - adenosine, R - purine, N - any base). The majority of Hfq-dependent sRNAs bind via the poly-U tails of their Rho-independent terminators to the proximal face and additionally to the rim (Class I sRNAs). This interaction protects the sRNAs from RNases and enhances their stability. Some sRNAs (Class II sRNAs) and most mRNAs bind to the distal site.^36^ A proposed mechanism is that Hfq with a prebound sRNA binds to ARN sites in the target, followed by mRNA scanning to find a stable sRNA/mRNA interaction site.^37^ Additionally, Hfq can also change RNA structures and resolve otherwise blocked interaction sites upon binding.^38^ As a general RNA-binding protein, Hfq can also bind to regulatory sites in mRNAs to modulate translation without an additional sRNA.^39^ In most instances, the RNA binding of Hfq is highly dynamic and RNAs need to compete for the existing Hfq molecules.^40,41^ Most research on Hfq was done in and , but Hfq is also important in many other Gram-negative bacteria.^42,43^ However, it is lacking in some Gram-positive bacteria and, where it exists, its role in RNA-based regulation is discussed controversially.^44^ In cyanobacteria Hfq likely does not even bind to RNA at all.^45^ Besides Hfq, other RNA chaperones have been discovered over the years, and how the increasing knowledge on RNA chaperones will feed into the design of synthetic sRNA-based regulators remains an exciting topic.

sRNAs are structurally modular and can be dissected into functional (seed region(s)) and structural (scaffold) elements which facilitate intracellular interactions and RNA stability. The synthetic biology principles of standardizing and characterizing individual elements in order to perform “plug-and-play” biology are applied to sRNAs, resulting in an enhanced sRNA understanding and the generation of improved regulators.^40,46,47^ However, in reality, sRNA-regulator-design relies to a high extent on trial and error because many aspects of RNA-based regulation are hard to predict. Typically, a range of candidates with seed regions complementary to the target are tested, and often designed manually. Normally this results in the detection of at least one functional synthetic sRNA, but in many cases it is not evident why different, equally promising-looking candidates yield very different levels of regulation. Sometimes the approach fails and no efficient sRNA regulator for a particular target is found at all, resulting in an unchanged phenotype of the sRNA-expressing strain (our unpublished data). Possible reasons for such failure include: (1) secondary structures of target mRNAs that prevent interaction, (2) high expression levels of target genes that undermine repression by sRNAs, (3) residual activity of the depleted protein pool that prevents phenotypic changes, (4) depletion of the sRNA by binding to off-targets, or (5) that an interaction has no regulatory consequence. Unfortunately, these results are not usually followed up, but their detailed investigation would provide valuable insights into the underlying mechanism(s). sRNA-based regulation is dependent on a multitude of factors and parameters which are likely only partly understood. For individual (natural) sRNA/target pairs the exact mechanisms are well investigated which allows extrapolation of general rules. However, despite decades of research, these examples remain anecdotal in the view of thousands of different UTRs within a single species, which hampers rational design for any given target of choice and complicates translation to other species. A truly generic design tool requires the basic and mechanistic understanding of all relevant factors. The advent of deep-learning based models might result in a breakthrough if used in a Laboratory 2.0. The Laboratory 2.0 uses standardized, highly characterized and interchangeable biological parts, enabling most processes to be automated, including data analysis, enabling researchers to spend the majority of their time on research rather than repetitive labor.^48^ This approach combines the biological and engineering sciences, implementing the Design-Build-Test-Learn (DBTL) cycle to facilitate high-throughput discovery and knowledge acquisition.^49,50^ Synthetic biology could provide the required high-quality training data for AI: thousands of available sRNA/target pairs. AI-models can most likely generalize the results obtained, to allow the understanding of underlying mechanisms, providing powerful design tools in the future. sRNAs are an ideal testbed to facilitate developments in robotics and AI and will have improved functional regulators as a measurable output. In the following section, we describe the current mechanistic understanding of sRNA-based regulation in bacteria and how this could be utilized for the design of synthetic sRNA regulators.

## Knobs and Levers for the Rational Design of sRNAs

Understanding sRNA-based regulation requires accounting for the in vivo complexity of these interactions. Figure 2 illustrates a comprehensive model (adapted from Reyer et al.^24^) showing key steps of sRNA regulation. Transcription of mRNA is separated into initiation (*k*_init_) and elongation (*k*_elon_), allowing for cotranscriptional events. The nascent precursor mRNA (mRNA_pre_) can either undergo sRNA-independent cotranscriptional decay (β_mpre_) or bind to an sRNA at rate *k*_on_. Both free and sRNA-bound mRNA precursors can be elongated, producing mature mRNA or mRNA/sRNA complexes, respectively. The sRNA/mRNA complex may be codegraded (β_ms_)^51^ or undergo sRNA-dependent cotranscriptional processing or termination (*k*_process_, *k*_term_ summarized in *k*_coreg_), producing a truncated, nonfunctional mRNA (mRNA_trunc_/sRNA). Importantly, the sRNA can be recycled from this complex via dissociation (*k*_off_). The sRNA is synthesized at rate α_s_ and degraded at rate β_s_. These molecular interactions regulate protein abundances by influencing mRNA stability, transcriptional termination, or translation efficiency. Regulation outcomes vary depending on factors such as target-specific features, interaction sites, and involved protein cofactors.

**Figure 2:**
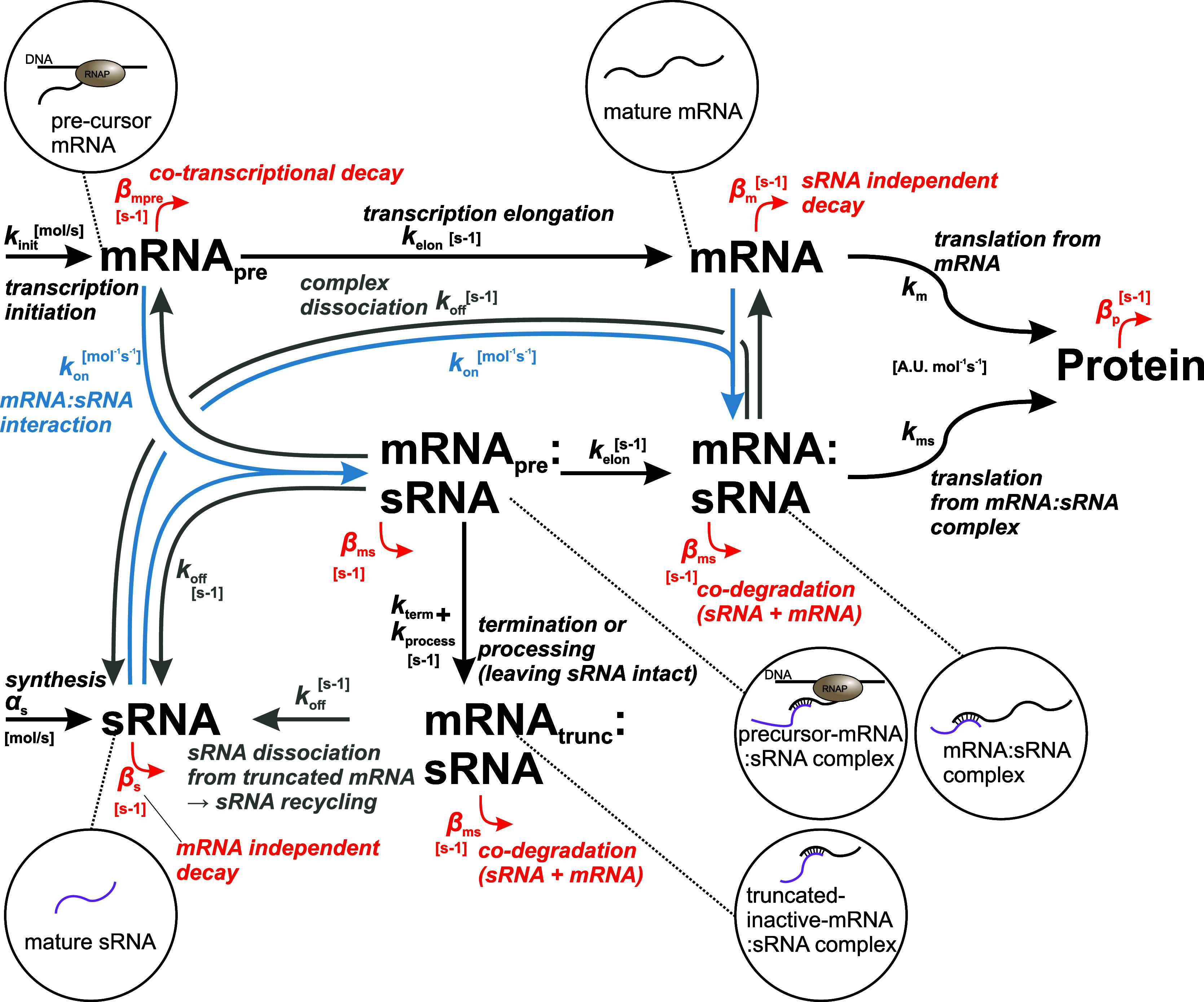
Scheme of sRNA-mediated regulation. Schematic of the sRNA-mRNA interaction model as described in the main text. The main adaptions to the previously published model^24^ are, that we explicitly include an mRNA_trunc_/sRNA complex and the dissociation of the sRNA from this complex, in order to illustrate the sRNA recycling. In the highlighted previous study this was implicitly handled via the precursor-mRNA/sRNA complex. In addition, we introduced an sRNA independent cotranscriptional decay and that the sRNA bound nascent RNA (mRNApre/sRNA) can be elongated to the mature complex.

### Global Sensitivity Analysis of Regulatory Strength to Regulation Parameters

To assess the regulatory potential of the sRNA-based regulation system described above (Figure 2) and most important the actual influence of specific parameters on the protein regulation strength, we performed a global sensitivity analysis (GSA) by simultaneously varying the model parameters. The results (Figure 3a) indicate strong interdependencies among parameters, with *k*_on_ (sRNA-target association rate) emerging as the most influential parameter both directly and overall. The stability of mRNA species (β_m_ & β_ms_), sRNA availability (α_s_), complex dissociation (*k*_off_), and translation rates (*k*_m_, *k*_ms_) all play important interactive roles, while parameters related to the basal synthesis and elongation of mRNA (*k*_init_, *k*_elon_), as well as cotranscriptional decay of premature mRNA (β_mpre_) and protein decay (β_p_), appear less impactful.

**Figure 3:**
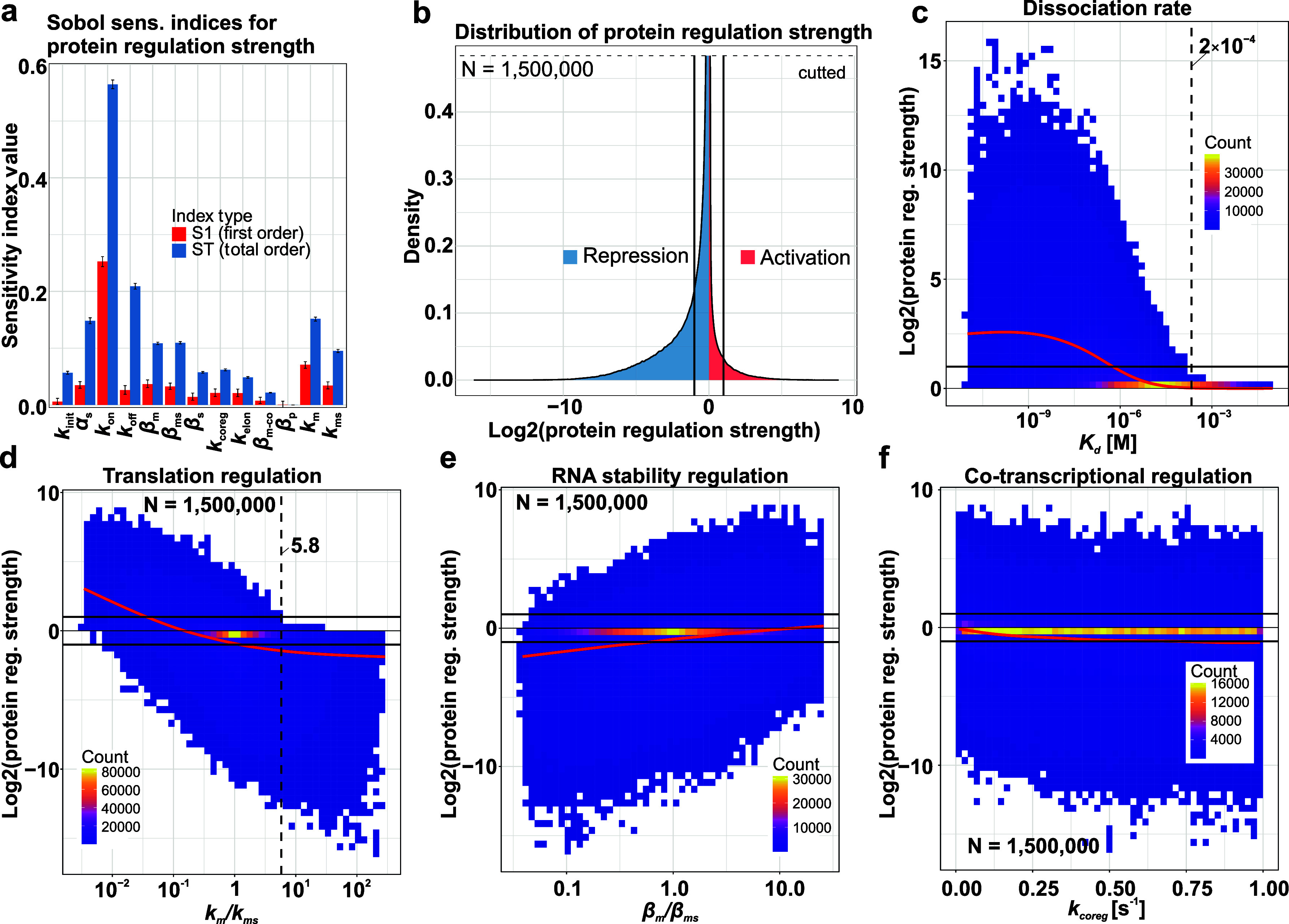
| Global Sensitivity Analysis of protein regulation strength. (a) First-order (S1, red) and total-order (ST, blue) Sobol sensitivity indices for key model parameters affecting protein regulation strength (defined as log 2 [*p*_scenario_/*p*_k_on=0_]). Protein regulation strength was defined as the steady-state log 2 ratio of the protein concentration with the sRNA and without, i.e., *k*_on_ set to 0. First-order Sobol indices (S1) quantify the direct contribution of each parameter to the output variance, while total-order indices (ST) account for both the direct effect and all higher-order interactions involving that parameter. Parameters were sampled as follows: *k*_init_ (uniform, 0.1–2), α_s_ (uniform, 0.1–2), *k*_on_ (log-uniform, 1.66 × 10^–6^—0.166 molecules^–1^ s^–1^ or 10^3^ - 10^8^ M^–1^ s^–1^ assuming a cellular volume of 10^–15^ L), *k*_off_ (log-uniform, 3 × 10^–2^ −100), β_m_, β_s_, β_ms_ and β_mpre_ (log-uniform, 0.0023–0.06 s^–1^ referring to half-lives of ∼5 min to ∼10 s), β_p_ (log-uniform, 5 × 10^–4^—0.05 s^–1^), *k*_m_ and *k*_ms_ (uniform, 0.1–30 AU × molecule^–1^ s^–1^). Error bars represent 95% confidence intervals from 100 bootstrap replicates based on 1,500,000 total model evaluations. *k*_on_ exhibits the largest direct (S1) and total (ST) influence, while parameters such as *k*_ms_, *k*_m_, β_m_, β_ms_, *k*_off_, and α_s_ show significant total influence predominantly through interactions (ST ≫ S1). Analysis was done with the “sensitivity” R-package. The code is available via GitHub (https://github.com/JensGeorg/GSA-sRNA-RNA-interaction-.git) and the respective data at zenodo (https://zenodo.org/records/15599898). (b) Distribution of protein regulation strength from *N* = 1,500,000 GSA samples. The distribution is sharply peaked around zero (no net regulation) but shows a significant skew toward repression (log 2 ratio <0), with smaller tails indicating potential for activation (log 2 ratio >0). Lines indicate 2-fold repression/activation (log 2 ratio = −1 or +1). (c–f) 2D binned density plots illustrating the relationship between specific parameter ratios or values (*x*-axis) and protein regulation strength (*y*-axis, log 2­[ratio]) from *N* = 1,500,000 GSA samples. Color intensity (dark purple to yellow) indicates the count of GSA samples within each bin. Red lines show the smoothed conditional mean using a GAM smoother. Black lines indicate ± 2-fold regulation as in (c). (c) Impact of the dissociation constant (*K*_d_ M) on the magnitude of regulation (absolute log 2 ratio). (d) Impact of the ratio of translation rates (*k*_m_/*k*_ms_) on regulation strength. (e) Impact of the ratio of key mRNA decay rates (β_m_/β_ms_) on regulation strength. (f) Impact of the cotranscriptional regulation/processing rate (*k*_coreg_) on regulation strength.

The distribution of protein regulation strength across 1.5 × 10^6^ GSA samples (Figure 3b) reveals a high frequency of outcomes near zero (log 2­[ratio] ≈ 0), indicating weak net regulation under many sampled parameter combinations. However, the distribution exhibits significant tails extending into strong regulatory regimes, and is notably skewed toward repression. Although sRNA-mediated regulation can yield a diverse spectrum of outcomes, there is a clear tendency for repression in the explored parameter space.

Analysis of parameter interdependencies revealed how specific relationships drive regulatory outcomes (Figure 3c–f). Stronger sRNA-target affinity (lower *K*_d_) correlates with stronger protein repression. Importantly, above a *K*_d_ threshold of ∼2 × 10^–4^, no parameter combination could achieve greater than 1.5-fold regulation. The translation rate ratio (*k*_m_/*k*_ms_) is also critical; high ratios favor repression, while low ratios can shift outcomes toward activation, though no activation was observed above a ratio of ∼5.8. Additionally, a less stable sRNA-mRNA complex (low β_m_/β_ms_ ratio) enhances repression, while cotranscriptional processing (*k*_coreg_) acts as a significant, rate-dependent repressive mechanism. More generally, this analysis shows that while extreme values for a single parameter can strongly favor a particular outcome (e.g., repression at low β_m_/β_ms_ ratios), the final regulatory strength is determined by the complex interplay of all parameters and even at extremes, a spectrum of regulatory strengths is possible.

### Kinetics of Regulation

Beyond steady-state outcomes, short- and midterm dynamics are crucial. The SgrS/*ptsG* system was analyzed during the first 24 min postinduction, based on experimental data.^24^

Models relying on codegradation and translational repression (Figure 4, gray lines) or only translational repression (Figure 4, red lines) proved insufficient. Both allowed protein levels to increase too fast and the model incorporating codegradation and translational repression additionally predicted an overly rapid sRNA decay. The best fit to experimental data was achieved by a model that included cotranscriptional regulation and sRNA recycling (Figure 4, blue solid lines). Removing sRNA recycling from this model again caused rapid sRNA depletion (Figure 4, blue dotted lines). As this analysis focuses only on a single system, the kinetics of other parameter sets can be explored in the accompanying Shiny app (https://jensrna.shinyapps.io/sRNA_RNA_int_app/).

**Figure 4:**
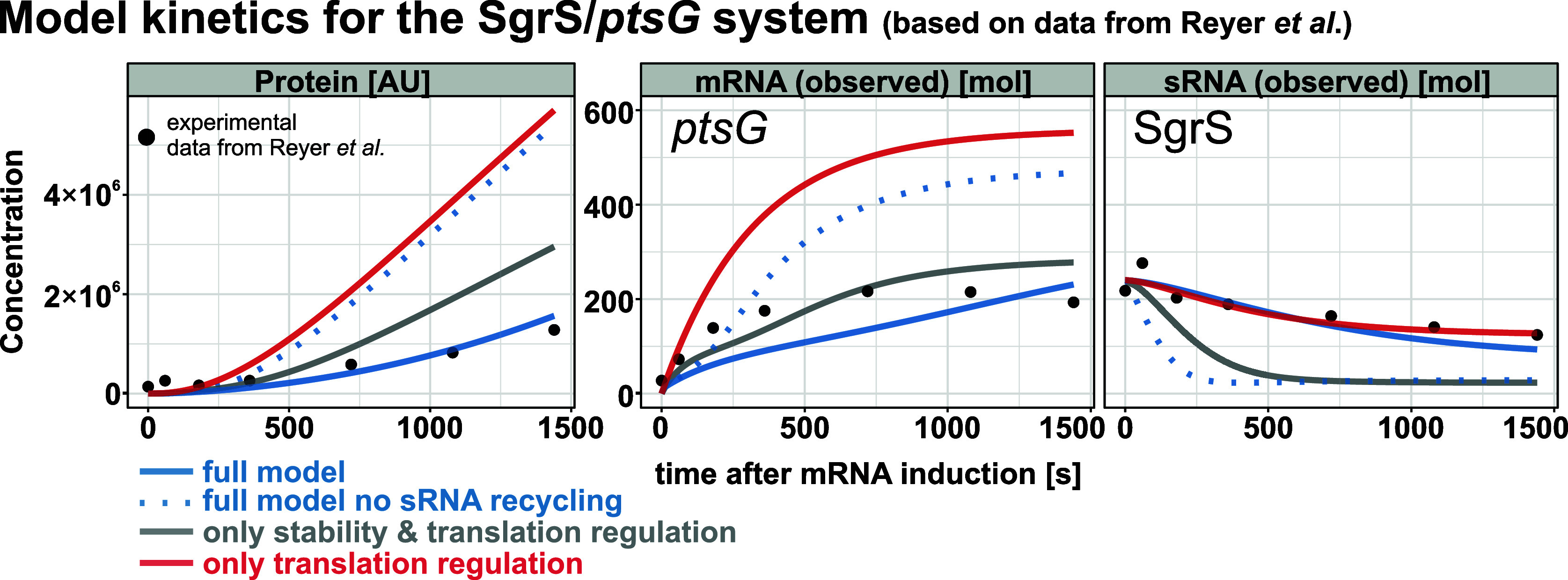
Comparative kinetic analysis of protein, observed mRNA, and observed sRNA concentrations under various experimental conditions. Simulations were carried out with the model displayed in Figure 2. Default parameters were: *k*_init_ = 1.8, α_s_ = 0.36, *k*_elon_ = 0.064, *k*_on_ = 0.0022, *k*_off_ = 0.3, β_m_ = 0.0032, β_s_ = 0.0015, β_ms_ = 0.0063, β_mpre_ = 0.0032, β_p_ = 0.00018, *k*_coreg_ = 0.68, *k*_m_ = 14 and *k*_ms_ = 1.96; based on Reyer et al.^24^ Changes to the default are stability & translation regulation (*k*_coreg_ = 0, β_ms_ = 0.02, *k*_ms_ = 0.18, *k*_init_ = 1.2); only translation regulation (*k*_coreg_ = 0, β_ms_ = 0.0032, *k*_ms_ = 0); in the full system without recycling, the dissociation of the sRNA from the mRNAtrunc/sRNA complex was not allowed. For the RNAs the assumed observed values are displayed, i.e., the sum of free and complexed molecules. The black dots represent the original experimental data from Reyer et al.^24^ Gray: System without cotranscriptional control. In order to better fit the data, the mRNA initiation rate has been reduced and the terms for translational and stability regulation have been increased. Blue: Full system with all regulation types (solid lines). System without the recycling of the sRNA from the terminated mRNA_trunc_/sRNA complex (dotted lines). Red: System with only translational regulation.

### Design Principles for Synthetic sRNA Regulators

This section connects the above regulatory theory to experimental practice by describing native processes and demonstrating how they can be harnessed for synthetic design.

### Target Translation

The most obvious sRNA-based regulation mechanism is the direct sRNA binding to the RBS of its target mRNA(s), which sterically interferes with translation initiation (TI) by the 30S ribosome. This direct inhibition works roughly in a five-codon window counted from the start codon^47,52^ which correlates with the mapped contacts of the 30S ribosome to mRNAs in vitro (reviewed in^52^). In addition, some indirect manipulations of TI through interactions outside the direct regulation window have been reported and further mechanisms might be discovered in the future. Binding of an sRNA too far upstream (e.g., Spot42/*sdhC* ^53^) or downstream of the RBS (e.g., SgrS/*manX* ^54^) for a direct inhibition may stabilize Hfq binding, which then sterically interferes with TI. Alternatively, sRNAs can inhibit translation by masking regulatory motifs, such as enhancers or ribosome standby sites within the target UTRs, or by preventing the translation of upstream open reading frames (uORFs) and leader peptides (reviewed in^55^). Design constraints: Direct inhibition of TI is a straightforward, and the most widely applicable, design principle for synthetic sRNAs. The actual TI window might vary for different target sequences and the seed region + sRNA scaffold combinations, but targeting the Shine-Dalgarno region and the start codon is a valid hypothesis to obtain a functional regulator. Constraints that might prevent choosing the optimal interaction site are (1) A seed region complementary to the direct inhibition window interferes with the secondary structure of the chosen scaffold. This could reduce the accessibility of the seed region resulting in a low binding rate, reduce the stability of the sRNA, and/or reduce the affinity to accessory factors. (2) An sRNA interaction with the seed region has an insufficient interaction energy, e.g., due to a high A/U abundance which results in a low binding rate. (3) The (natural) accessibility of the chosen target region is low (e.g., through secondary structures). Unlike direct TI inhibition, translation activation and the described indirect negative mechanisms require additional features in the target, making a generic one-size-fits-all approach impractical. However, if specific features of the mRNA of interest are known through individual studies or high-throughput methods - such as Ribo-seq for the detection of uORFs or ribosome pausing sites^56^ - the seed region can be selected accordingly. There may be other aspects influencing the design of optimal sRNA regulators. Recent prediction programs show that a rational approach for the design is feasible.^57–59^ However, further improvements are needed which may be achieved with a combination of high-throughput data generation and AI.

### Target RNA Stability

RNA decay in bacteria involves endoribonucleases and exoribonucleases. The textbook RNA-decay is initiated by an endonucleolytic cleavage (mostly RNase E), and the resulting decay products are then further digested by 3′ to 5′ exonucleases.^60^ Two modes for RNase E dependent decay are discussed. In the 5′ entry mode, RNase E detects 5′ monophosphate and initiates decay at sensitive internal sites starting from the 5′ end. This mode is supported by the finding that 5′ triphosphates inhibit RNase E dependent decay. However, there is also a 5′ independent direct entry mode, where sensitive internal sites are directly targeted (reviewed in^60^). sRNA binding can increase the decay rate by active recruitment of the degradosome or RNases^21,23,61^ as well as by an indirect effect of the translation inhibition, where the lack of translating ribosomes exposes RNase sites in the mRNA and promotes decay.^62^ Despite this indirect effect, translation inhibition and enhanced decay are separated in some examples. In , MicC binds *ompD* mRNA starting 67 nt downstream of the start codon, which does not affect translation but triggers RNase E dependent decay.^63^ In contrast, some sRNA/mRNA interactions only affect the translation rate but not the decay constant (e.g., Spot42/*sdhC*,^53^ RprA/*hdeD* ^64^), while interactions of the same mRNAs with other sRNAs do trigger degradation (e.g., RyhB/*sdhC* & RybB/*sdhC*,^53^ CyaR/*hdeD* ^64^) by active recruitment of RNase E. The interaction sites of the above highlighted examples are not identical, but it is worth considering that the observed differences might be due to the involved sRNAs and some sRNA scaffolds might be more prone to recruit the degradosome than others.^46^ For MicC it was even shown that the sRNA can allosterically activate RNase E.^65^ The mechanism of RNase E activation by sRNAs such as MicC, RyhB, CpxQ and SroC is not entirely understood but might facilitate the direct entry mode of RNase E as it seems to be independent of the 5′ phosphorylation state of the sRNAs,^61^ which contrasts previous hypotheses.^65^ However, it was shown that the 5′ phosphorylation state can have a strong sRNA-specific influence on the sRNA stability itself.^61^ The decay also likely involves recruiting additional factors such as Hfq to the C-terminus of RNase E.^61^ sRNA binding can also mask RNase sites and thereby increase the stability of the bound mRNAs. An example is the stabilization of *cfa* mRNA by a RydC/Hfq complex.^66^ In general interactions of sRNAs with the RNase E dependent degradosome are most widely investigated, but also recruitment of the double strand specific RNase III has been described,^67^ and other RNases might be additionally involved. Design constraints: achieving directed, rational control over target stability would require in-depth knowledge of its natural degradation pathways, and has, to the authors’ knowledge, not been actively pursued in synthetic biology. Designing such control would require selecting an appropriate interaction site and an sRNA scaffold known for susceptibility to RNase-mediated repression or activation. For instance, RNase E cleavage sites, which can be globally identified by TIER-seq,^68^ could potentially be masked or enhanced by an engineered sRNA. Some potential targets may not be addressable, as only approximately 60% of RNAs are initially degraded by RNase E, with another 15% processed by other known RNases. This leaves the mechanisms initiating decay for the remaining 25% of RNAs still unknown.^60^ Also, due to interference from actively translating ribosomes, regions outside the 5′ UTR may be less accessible to sRNAs in general.

### Target RNA Synthesis Rate

The maximal possible synthesis rate of an mRNA is defined by its promoter, but transcription termination sites in the 5′ part of the transcript can reduce the synthesis rate of the full-length transcript. sRNAs can affect the synthesis rate of their target mRNAs by modulating this premature Rho-dependent or Rho-independent termination. Evidence from examples such as ChiX/*chiP*,^69^ OppZ/*oppB*,^70^ Spot42/*galK*,^25^ and SgrS^24^ suggests a shared mechanism for Rho-dependent termination control: sRNA-mediated inhibition of translation exposes Rho utilization (Rut) sites in the mRNA. This exposure subsequently promotes termination and reduces the mRNA synthesis rate as a secondary effect.^71^ In the opposite case, sRNAs mask Rut sites in the 5′ UTR of their targets, thereby preventing premature transcription termination in order to activate target mRNA expression.^71^ Regulation of Rho-independent termination requires the presence of potential terminating secondary structures in the mRNA for which the folding can be modified by sRNA binding. An example is the *mnrS* riboswitch in which terminates transcription in the absence of manganese and folds in a nonterminating structure in the presence of manganese. The binding of the sRNA IsrR to the riboswitch sequence traps it in its terminating state.^72^ Design constraints: Direct control of the synthesis rate by synthetic sRNAs is difficult and requires knowledge about potential Rut sites or terminating secondary structures in the target UTR. However, it was shown in that Rho is an attenuator of hundreds of genes,^26^ making this an interesting theoretical opportunity for the activation of the respective genes by sRNAs.

### Binding Affinity and Kinetics

The exemplary analysis (Figure 3) highlights that the sRNA-target interaction is the crucial prerequisite and a key parameter for sRNA-based regulation. The most important, though not the only, determining aspect is base pair complementarity. Besides the complementarity, the interaction regions need to be accessible in both sRNA and target. The association and dissociation constants (*k*_on_ & *k*_off_) are also dependent on RNA-binding proteins (RBPs) such as Hfq. Hfq sites in the mRNA clearly facilitate the binding of an sRNA/Hfq complex which increases the association rate.^21,37,73^ Binding and regulation without Hfq is also possible, although this might require higher sRNA concentrations or stronger complementarity. The use of natural sRNA scaffolds increases the probability that binding requirements for relevant host-specific RBPs are met. In general, base pair complementarity determines the target and the specific interaction site. Natural sRNAs often have multiple targets and typically pair through short, imperfect stretches of complementary bases. In the authors’ view, the matching of endogenous sRNAs has evolved over a long time, ensuring that only desired sequences are regulated and nonbeneficial off-targets are avoided. Presumably, this evolution mostly took place in the mRNA UTR sequences and not in the seed region of the sRNA, allowing an sRNA to “collect” multiple targets. Therefore, the rational design of multitarget seed regions for user-defined endogenous targets without the natural long-term co-evolution will result in ineffective regulation and/or off-target effects (own unpublished results). Design constraints: the choice of the seed sequence with a strong complementarity to the desired region in the target mRNA, without interfering with the scaffold structure and without complementarity to other mRNAs (unwanted off-targets) is a major step in the rational design of an RNA-based regulator.^57^ Consequently, the seed sequence defines the target and the interaction site. Computational tools can help to find complementary stretches with a high binding affinity and accessibility in the region of interest. To avoid off-targets, they can also check if the seed-sequence has a noteworthy complementarity to other UTRs in the genome. In theory, imperfectly matching seed regions could also be designed; however, it remains unexplored whether such designs would have any different regulatory effect, and, if so, whether these effects would be beneficial or detrimental. Despite the existing tools, the rational design of sRNAs is hindered at this step because the available bioinformatic tools for structure and interaction prediction provide at best an approximation of the biological reality. Furthermore, the predicted thermodynamic hybridization energy is not always a good proxy for the actual molecular kinetics.^24^

### Brief Summary of Synthetic Regulator Design Aspects to Consider for In Vivo Application

The above-described mechanisms require stoichiometric binding of sRNA and target, thus the available concentration of the sRNA in relation to the mRNA concentration is also important. Recycling of the sRNA is conceivable if it is not codegraded with its target or if it dissociates after a successful termination event (Figure 2).^24^ The available sRNA concentration is dependent on its transcription rate, decay constant, and potential binding to other (off)-targets and RNA sponges that could reduce the availability for the target of interest. The sRNA synthesis rate is easily controllable by the choice of the promoter. The sRNA stability is less predictable, but the design process should ensure that the selected seed region does not introduce RNase cleavage sites^46^ that would reduce the stability of the synthetic sRNA. In addition, the selected seed region should not interfere with the scaffold in order to maintain the overall structure and stability of the sRNA. However, in theory, a targeted modification of the sRNA stability could also be of use in some contexts. So far there are no ways to a priori predict the stability of a newly designed regulator and the actual concentration and stability must be checked experimentally.

## Construction of Synthetic sRNA Transcription Units

In order to create synthetic sRNAs, it is still necessary to use the structural elements of natural sRNAs as templates (Figure 5a). sRNAs can be identified from bacterial genomes using transcriptome data, which also enables annotation of their sequences.^74^ The initial step to use a natural sRNA as a template for synthetic sRNAs is to predict its seed region (which can, for example, be done with IntaRNA (CopraRNA)^75^ or SPOT^76,77^ ), which then allows for alteration of the sequence to be tailored to a specific target sequence for its direct application (Figure 5b). This has been accomplished on numerous occasions, as evidenced by the alteration of phenotypes or the control of metabolic fluxes.^40,47,78,79^ In this context, it is crucial to consider the most important design constraints including the ones mentioned in the previous section: (1) the seed region must be accessible and should not be sequestered in an internal RNA structure; (2) the overall structure of the RNA should not be disrupted; (3) the seed sequence must be highly specific to avoid off-target effects; and (4) the choice of target site, and potentially the scaffold itself, influences the regulatory mechanism and the regulation strength, as discussed above. In the absence of additional information, a repressive seed design typically targets the RBS and the start codon.^40,47,58^ Activation requires either known inhibitory elements in the UTR that can be counteracted or additional genetic engineering within the UTR. In some cases, it may not be possible to optimize all factors simultaneously, necessitating a trade-off between design aspects. Some computational tools have been developed to address these design constraints and facilitate the generation of synthetic sRNA sequences on a global scale.^57,59^ However, there is still room for improvement and an interesting alternative to traditional methods are the emerging deep-learning tools for 2D and 3D RNA structure prediction.^80,81^ AlphaFold 3 even allows modeling of RNA/RNA interactions together with accessory RBPs. While the accuracy of such models is hard to evaluate, the future prospect to evaluate the influence of different seeds and RBPs on the structure with the emerging deep learning models is fascinating. In general, current and future design tools return a list of sequence strings which can be narrowed down by their in silico characterization using predictive tools to obtain a reasonable number of sequences. The sequences ultimately need to be synthesized as DNA and functionally validated in vivo by heterologous expression prior to their targeted application.

**Figure 5:**
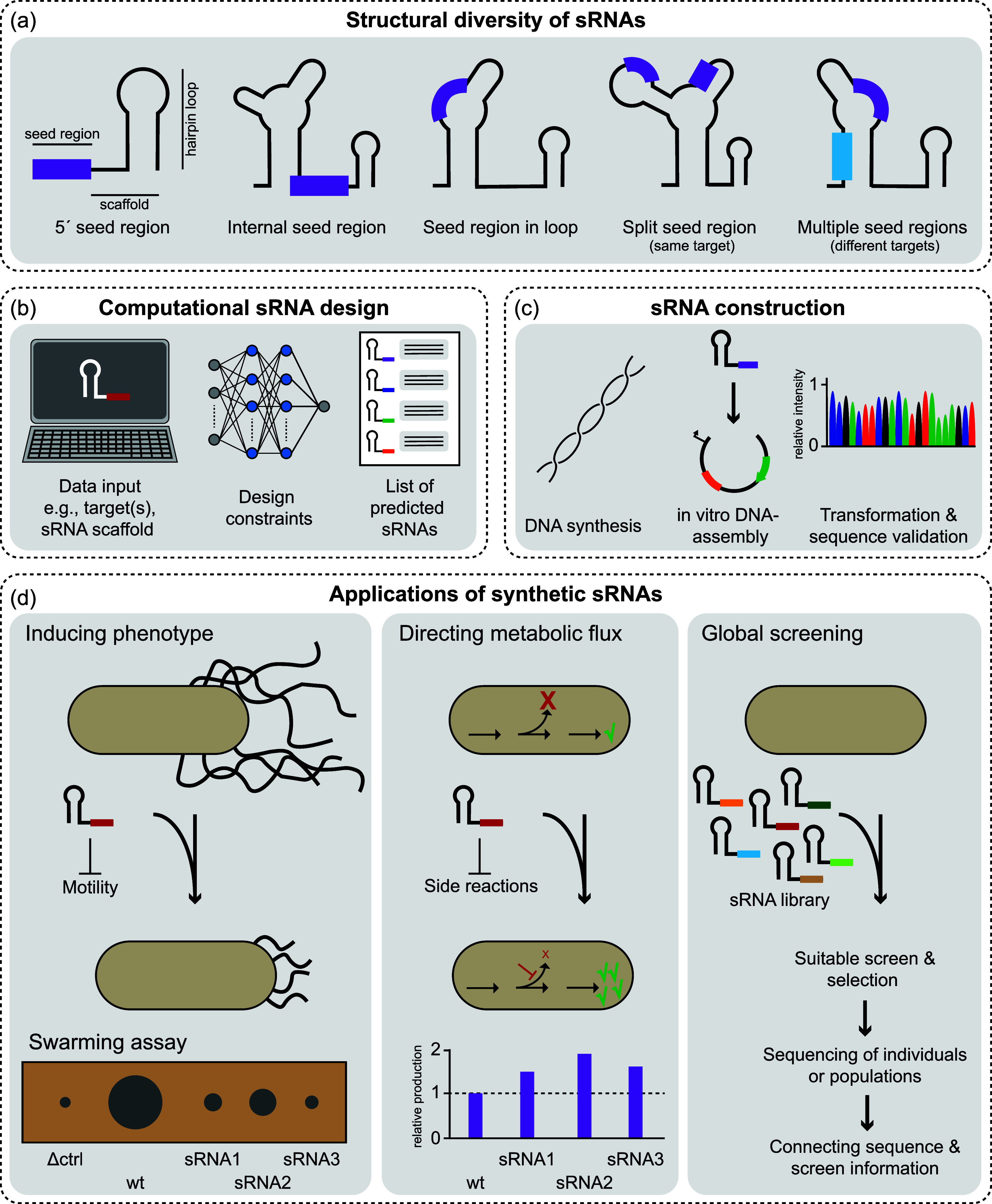
Design, construction and application of synthetic sRNAs. (a) Illustration of the structural diversity and seed region (purple and blue) location(s) of sRNAs. The core elements of an sRNA are the scaffold and the seed region. (b) Computational tools allow for the systematic design of synthetic sRNAs, taking into account multiple design constraints (e.g., target site, off-target sites, sRNA structure) and returning a list of DNA sequences for DNA synthesis. (c) sRNAs are short sequences and can be synthesized either as double-stranded DNA or as oligonucleotides, which are subsequently used for in vitro DNA assembly. The DNA assemblies are validated by sequencing after transformation. (d) Synthetic regulators have a wide range of applications. They can be used to induce phenotypes to study biological functions or to control metabolic fluxes for improved production. Due to their small size and versatility, sRNAs are well suited for global libraries in basic and applied sciences.

Significant advances in DNA synthesis and assembly have been made and in particular the advent of enzymatic DNA synthesis will enable the generation of highly complex and repetitive DNA sequences which is currently still a challenge for many DNA synthesis providers.^82^ The general technological advances for DNA synthesis and assembly have recently been reviewed.^82–84^ This article focuses on recent trends to build synthetic sRNAs in bacteria (Figure 5c). DNA sequences encoding synthetic sRNAs can be cloned into expression plasmids or integrated into the genome to study their effect in vivo with the method of choice. Expression plasmids provide simple and rapid platforms to assess the functionality of generated sRNAs for their application in contrast to more complex integration into the genome.^47^ Based on their small size nearly all DNA assembly strategies are applicable to construct expression cassettes. In particular, Gibson Assembly^85–87^ and standardized methods such as Golden Gate cloning^88^ and the hierarchical Modular Cloning (MoClo)^89^ are popular because they allow for rapid and versatile construct generation.^46,47^ MoClo is based on Golden Gate cloning and allows the generation of reusable libraries of parts for the construction of transcription units (TUs) and the subsequent assembly of multi-TU constructs. In the end, the in vitro DNA assembly needs to be reliable and suitable for the experimental environment. Initially setting up a MoClo compatible workflow with the respective basic parts and acceptor plasmids can be cumbersome. Nevertheless, MoClo becomes highly rewarding once respective part libraries are established or are available from the community. Multiple systems have already been established but no sRNA-specific collection is available yet. For synthetic sRNAs in particular, standards with minimal scar sequences are highly useful for construction because sRNA structures can be affected by a single nucleotide.^46,58,90^

## Testing Synthetic sRNAs and Learning from the Obtained Results

Recent studies have cherry-picked a few synthetic sRNAs that work for their intended application, but no systematic testing information has been made available to the community. In particular, in the light of automated laboratories, large data sets are produced and tested but often they are not accessible. However, this becomes increasingly important in ML and AI-based analysis. Information about the regulators that do not work, and their mechanistic details, are highly relevant, but negative results are usually not published and even more rarely followed up on. Learning (in synthetic biology) is the process of acquiring new knowledge/understanding based on data - including negative outcomes. Testing is an incremental process in engineering in order to build and improve a functional system. The efforts of standardization in synthetic biology create increased compatibility of biology with systematic approaches which can be applied to sRNAs. This allows for laboratory automation workflows to design, construct, test, and apply large numbers of constructs in parallel.^91^ Pooled sRNA libraries may offer advantages for the development of synthetic sRNAs, their investigation in biological systems or their application (Figure 5d).^46,92^

Once synthetic sRNAs are constructed, the first step is to test them for their functionality. The actual function is defined by the user’s experimental design and implemented by selecting a seed region to regulate the desired target (examples are shown in Figure 5d). Validation of functionality by testing for a phenotype is quick and straightforward to assess in a qualitative and/or quantitative manner, for example, motility^93^ or resistance mechanisms.^92^ Based on the results obtained, conclusions can be drawn as to whether functionality is sufficient or whether design parameters need to be adjusted. The testing phase can be performed in high throughput using automated and library-based approaches, rapidly generating data points to draw conclusions about sRNA functionality.^46^ However, often applications may not allow for simple read-outs (e.g., phenotype, pigment accumulation), resulting in complex downstream analytics which may not be accessible in high throughput or which are associated with high costs. To circumvent this, the function of a regulator should initially be tested and improved in a reporter assay (e.g., phenotypic or fluorescence assay). The data obtained from the testing phase feeds into the learn phase which, in engineering, results in the interactive Design-Build-Test-Learn cycle.

Based on recent studies, several aspects to be considered for synthetic sRNAs have been elucidated. Most importantly, it was shown that sRNAs are useful tools for regulating metabolic fluxes and controlling phenotypes in bacteria.^40,47^ Recent studies have shown that the construction of sRNAs is compatible with Golden Gate cloning and laboratory automation.^46,47,58,94^ This allows the construction of libraries of synthetic sRNAs to study, for example, the effect of seed region length.^46^ The results of this study include that a minimum length of 12 nt for the seed region should be considered, that a single nucleotide can change the structure resulting in strongly reduced regulation, and that AU-rich sequences lead to instability of the primary transcript due to RNase E cleavage. Such results can directly improve global prediction tools for optimized synthetic regulators. In turn, it suggests that more data are needed to better understand sRNA biology, allowing for improved sRNA-based regulators as molecular tools in basic and applied research. With recent developments (e.g., robotics in experimental biology) and interdisciplinary approaches, the time has come to generate large data sets to systematically study sRNA biology and harness sRNAs as synthetic regulators. The resulting data will enable AI and ML-based approaches to facilitate research and uncover potentially overlooked insights. Importantly, the majority of the learning aspects focus on the seed region and the ability to efficiently regulate the target and with this the phenotype. The initial application of sRNAs as a tool for metabolic engineering to improve production screened various different scaffolds to select the optimal ones.^40^ However, very little work has been done on the engineering of sRNA scaffolds to improve the regulators. A recent study highlights the transferability of sRNA technology to organisms lacking the Hfq protein by heterologous coexpression of sRNA and Hfq,^91^ which complicates the simplicity of sRNA-based regulators. Therefore, research should put emphasis on sRNA scaffolds to potentially identify sequences that have a broad host range and are highly stable. Natural scaffolds can be used as blueprints and adapted toward simple and robust structures. For example, scaffolds can be minimized to consist of only a short stem-loop and a poly-U stretch, essentially a simple transcription terminator to which a synthetic seed region is attached at the 5′ end. Such minimized sRNAs may function in an Hfq-independent manner and across species.^95^ Hypothetically, such minimized scaffolds even reduce unspecific activities in vivo (e.g., off-targets), but this needs to be addressed on a case-by-case basis. Modular synthetic biology, coupled with systems biology approaches, could provide the necessary data in native and heterologous hosts to uncover different aspects of sRNA scaffolds, providing the basis for AI-improved and broadly applicable sRNA-based regulators.

### Concluding Remarks and Future Perspectives

In this article we have highlighted the function of sRNAs, and their use as synthetic post-transcriptional regulators in prokaryotes. In contrast to CRISPR/Cas systems, sRNAs are much simpler and presumably cause less cellular burden. As described above, sRNAs designed to target multiple user-defined mRNAs with a single seed region are likely not feasible, as such a strategy would inherently result in a significantly reduced complementarity and interaction probability with individual targets (a factor that scales with the number of targets) and/or a high probability of off-target effects. Nevertheless, due to their small size, arrays of independent regulators can be designed, constructed, and applied, allowing the regulation of multiple targets in a precisely controlled manner. These arrays can be designed in multiple ways: as (1) a single TU resulting in an sRNA targeting multiple mRNAs,^96^ (2) a single transcript which is processed into individual sRNAs with individual targets,^29^ or (3) an array of multiple TUs.^47^ In addition, constructed sRNAs may be used across diverse bacterial species using established tunable systems.^29,91^ The coexpression of sRNAs and Hfq to achieve portability between different species seems to be a successful approach.^91^ One may argue that this would be similar to CRISPR/Cas systems, however, Hfq is a small protein of only 102 amino acids in size, potentially causing less burden to the cell in case of heterologous expression. Nevertheless, research could focus on identifying, characterizing, and improving Hfq-independent sRNAs with a broad host range as efficient cross-species regulators and the exploitation of host specific proteins and mechanisms.

In the future, research on synthetic sRNAs will increase. AI and ML-based design and data mining will become an important aspect. We anticipate that design and characterization of synthetic sRNA scaffolds will offer multiple opportunities. The research in this direction is currently limited, but initial studies are appearing.^97^ On the one hand, they will help to elucidate the basic mechanisms of sRNA-based regulation. Synthetic scaffolds may also allow the creation of orthologous highly stable or bistable regulators (e.g., combination of sRNA and RNA thermometer or sRNA and riboswitch). On the other hand, the high-throughput construction and characterization can drive developments toward large-scale RNA structure determination, which can be the basis for new methodologies to determine RNA structures in vivo in high throughput. From this perspective, sRNAs may not only serve as versatile and simple-to-program post-transcriptional regulators but also as a testing environment for the development of new technologies with a broad application spectrum.
